# Two new species of *Pterostichus* Bonelli subgenus *Pseudoferonina* Ball (Coleoptera, Carabidae, Pterostichini) from the mountains of central Idaho, U.S.A.

**DOI:** 10.3897/zookeys.104.1272

**Published:** 2011-06-13

**Authors:** James C. Bergdahl, David H. Kavanaugh

**Affiliations:** 1 Conservation Biology Center, University of the Wilderness, 919 S. Adams St., Spokane, WA 99204, U.S.A.; 2 Department of Entomology, California Academy of Sciences, 55 Music Concourse Drive, San Francisco, CA 94118, U.S.A.

**Keywords:** Coleoptera, Carabidae, *Pterostichus*, *Pseudoferonina*, *Melvilleus*, North America, Pacific Northwest, Idaho, Salmon River Mountains, Clearwater Mountains, flightlessness, endemism, montane streams

## Abstract

Two new species of *Pterostichus* Bonelli subgenus *Pseudoferonina* Ball, are described from the mountains of central Idaho: *Pterostichus bousqueti* Bergdahl [type locality = small tributaries of South Fork of Payette River watershed, ca. 1170 m (3840 ft), 44.0675°; -115.6822°, near Lowman, Salmon River Mountains, Boise County, Idaho, U.S.A.] and *Pterostichus lolo* Bergdahl [type locality = Cottonwood/Orogrande Creek, ca. 870 m (2850 ft), 46.5528°; -115.5522°, North Fork of Clearwater River watershed, Clearwater Mountains, near Bungalow, Clearwater County, Idaho, U.S.A.]. Males of *Pterostichus bousqueti* and *Pterostichus lolo* are easily distinguished from each other and the seven previously described *Pseudoferonina* species by the form of the median lobe of the aedeagus, and from most individuals of the other species of *Pseudoferonina* in Idaho by features of pronotal shape and macrosculpture. Both species appear to be obligate ripicolous hygrophiles, restricted in distribution primarily to the margins of small montane streams in forested areas. Widespread intensive stream surveys for *Pseudoferonina* over many years indicate the geographic ranges of both species are highly localized, and additional undescribed species may occur in Idaho.

## Introduction

*Pterostichus* Bonelli (1810) is one of the largest genera of carabid beetles in North American north of Mexico, including about 200 described species and subspecies, classified in about 20 species groups and at least as many recognized subgenera (Bousquet and Larochelle 1993; Bousquet 1999). They represent almost 8% of this region’s carabid fauna (ca. 2640 species and recognized subspecies). The *Pterostichus* subgenus *Pseudoferonina* Ball (1965) (type species = *Pterostichus lanei* Van Dyke 1925) is one of three *Pterostichus* subgenera narrowly restricted (endemic) to the Pacific Northwest (Washington, Oregon, Idaho and British Columbia), and the most diverse (Bergdahl 1995). Adults of all *Pseudoferonina* species are strict ripicoloushygrophiles whose habitat is the margin of small, forested headwater streams in mountainous terrain. They can be found from near sea level to subalpine elevations, but especially mid-montane zones where low (0–2) order streams (Strahler 1957) are common. None of the species are known to be alpine.

The carabid fauna of the Pacific Northwest [ca. 705 recognized species and subspecies (Bousquet and Larochelle 1993, Bergdahl 1995)] is reasonably well known. Most of the species were described many decades ago (mean date of publication = ca. 1865), and any remaining undiscovered species are probably very localized and difficult to find (e.g., Kavanaugh and Labonte 2006; Labonte 2006), or cryptic sibling species. *Pseudoferonina* is one of the least understood and most fascinating groups of closely related carabids in the Pacific Northwest. Over the last 26 years, 6 of the last 13 new carabid species to be described from the Pacific Northwest are members of subgenus *Pseudoferonina*.

*Pseudoferonina* includes six previously described species that appear to form a distinct group: *Pterostichus lanei* (Van Dyke 1925), *Pterostichus humidulus* (Van Dyke 1943), *Pterostichus shulli* (Hatc 1949), *Pterostichus campbelli* Bousquet (1985), *Pterostichus smetanai* Bousquet (1985), and *Pterostichus spathifer* Bousquet (1992) (Bousquet and Larochelle 1993). The subgenus also includes *Pterostichus vexatus* Bousquet (1985), which both Hatch (1953) and Ball (1965) misidentified as *Pterostichus shulli*. Males of *Pterostichus vexatus* differ from those of other members of the subgenus in having: 1) a short median lobe, with apical lamella flattened dorso-ventrally (not laterally), 2) the gonopore terminal, not basal, on the internal sac, 3) the internal sac everted dorsally, not ventrally,and 4) lackingthe semicircular indentation (flattening) of the last visible abdominal sternite that is distinct in males of all of the other species. Based on these features, Ball (1965) described a new subgenus for *Pterostichus vexatus* (i.e., his “*Pterostichus shulli*”), *Melvilleus* Ball, which is currently considered a subjective junior synonym of *Pseudoferonina* (Bousquet 1985, 1992, 1999, Bousquet and Larochelle 1993). Among species of *Pseudoferonina* found in Idaho, *Pterostichus vexatus* has the broadest geographical and habitat ranges.

The purpose of this paper is to describe two new species of *Pseudoferonina* from the Clearwater and Salmon River mountains of central Idaho, including information about form and structure, geographical and habitat distributions, and way of life. Hatch (1953) provided a key to the carabids of the Pacific Northwest that includes *Pterostichus lanei* and *Pterostichus vexatus*. Lindroth (1966) provided excellent keys to most of the subgenera and species of *Pterostichus* in the Pacific Northwest, but he did not include *Pseudoferonina*. Bousquet (1985) provided a key for identification of adults of the seven *Pseudoferonina* species known to him and later modified that key (Bousquet 1992). In this paper we suggest modifications to his key to accommodate the two new species we describe.

## Materials and methods

All specimens of the new species described here were collected by the first author [JCB] in 1999, 2009 or 2010. These specimens were acquired by timed hand collecting along small streams in mountainous terrain (e.g., Dupuis and Friele 2006), turning over rocks and organic debris primarily with a handheld garden fork, and flooding small gravel bars (Darlington 1971) using a plastic gold pan. Specimens were preserved in the field in a mixture of 70% ethanol + 30% white vinegar. As soon as possible in the lab, specimens were washed in warm water and then stored in fresh aliquots of the same ethanol/vinegar mixture to await preparation. Stream names and the names of other geographic features were derived from 1:100,000 United States Geographic Survey topographic maps and United States Forest Service forest district road maps. Geographical coordinates and elevations for collecting sites were determined using Google Earth (www.earth.google.com).

Specimens were examined using an 8–50x Nikon dissecting scope with a high-intensity light tube. The only measurement recorded was apparent body length (ABL), measured from the apex of the labrum to the apex of the longer elytron. Digital photographs of dorsal habitus and male genitalia were taken using an Auto-montage imaging system by Syncroscopy with a Leica M420 dissecting microscope.

Specimens examined in the course of this study are deposited in the following collections:

CAS California Academy of Sciences, San Francisco, California, U.S.A.

CMNH Carnegie Museum of Natural History, Pittsburgh, Pennsylvania, U.S.A.

CNC Canadian National Collection, Ottawa, Ontario, Canada

JCB James C. Bergdahl Collection, Spokane, WA, U.S.A.

OSU Oregon State University Arthropod Collection, Corvallis, Oregon, U.S.A.

UICM University of Idaho, Moscow, Idaho, U.S.A.

WSU Washington State University, Pullman, WA, U.S.A.

## Taxonomy

### Pterostichus (Pseudoferonina) bousqueti

Bergdahl sp. n.

urn:lsid:zoobank.org:act:1D065517-89A3-4A7F-8025-0522484E5CEF

http://species-id.net/wiki/Pterostichus_(Pseudoferonina)_bousqueti

**Figure 1.**
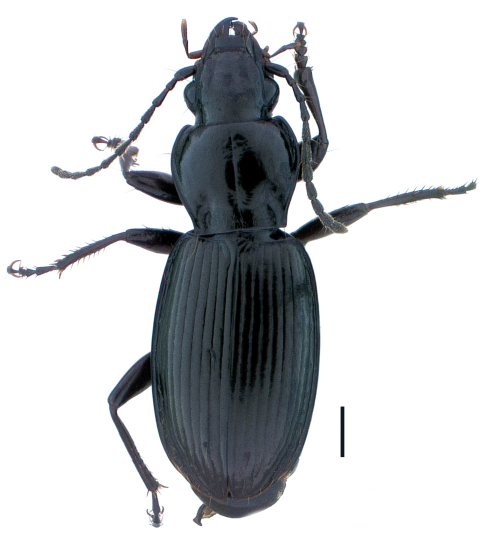
Holotype male, *Pterostichus (Pseudoferonina) bousqueti* Bergdahl, sp. n., dorsal habitus. Scale line = 1.0 mm. Automontage digital image by D. H. Kavanaugh.

Figs 1 3A 4A, 4C 5 6

#### Type locality.

U.S.A., Idaho, Boise County, small tributaries of the South Fork of the Payette River near Lowman, ca. 1200 m (3940 ft) elevation, 44.0675°; -115.6822°.

#### Type material.

Holotype: a male, deposited in CAS, labeled: “USA: Boise Co., Pine Flat Creek at South Fork Payette River Road, 6 km W of Lowman, 44°04'03"N; 115°40'56"W, T9N, R7E, S31, elev. 1160 m (3805 f), South Fork Payette River Basin, Boise National Forest, 05 May 1999, #26-1999, J. C. Bergdahl, coll.”/ “HOLOTYPE *Pterostichus bousqueti* Bergdahl designated 2011” [red label]. Paratypes: A total of 11, with 8 males and 1 female with the same data as the holotype, and 2 males labeled: “USA: Boise Co., Archie Creek near confluence with SF Payette R., 8 km E of Lowman, Boise Natn. Forest, 44°04'15"N; 115°31'06"W, T9N R8E S33, elev. 1240 m (4070 ft), South Fork Payette R. watershed, 06 May 1999, #33-1999, J. C. Bergdahl, coll.” The single female paratype is deposited in CAS, the male paratypes in CNC, CMNH, OSU, UICM, WSU, and JCB.

#### Etymology.

The specific epithet, *bousqueti*, is a Latinized version (genitive case) of the surname of Dr. Yves Bousquet (Agriculture Canada, Ottawa, Ontario, Canada), in recognition of his outstanding contribution (Bousquet 1985) to our knowledge of *Pseudoferonina*, which brought order to the chaos in taxonomy of the species known at that time.

**Figure 2.**
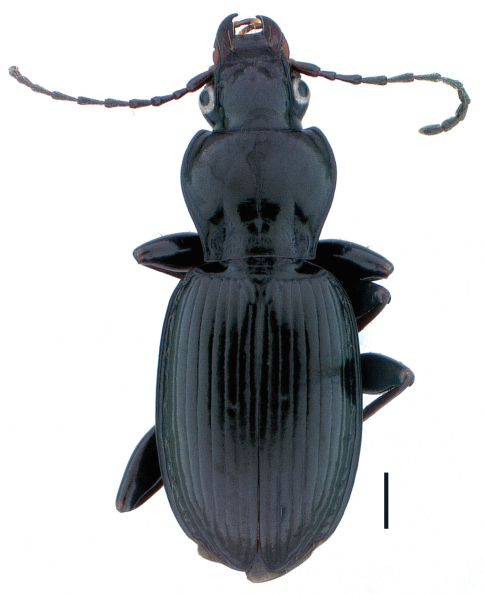
Holotype male, *Pterostichus (Pseudoferonina) lolo* Bergdahl, sp. n., dorsal habitus. Scale line = 1.0 mm. Automontage digital image by D. H. Kavanaugh.

#### Diagnosis.

A *Pterostichus* with the characteristics of members of subgenus *Pseudoferonina* (see Ball 1965, and Bousquet 1999); males easily distinguished from those of all other species of *Pseudoferonina* by the shape of the median lobe of the aedeagus, especially the apical lamella (apex), which is shaped (in left lateral view) like the blade of a hatchet or tomahawk (Figs 4A and 4C). Also, the shaft of the median lobe in lateral view is thicker in the middle than at either end (where it joins to the base or the apical lamella) and lacks the distinct mid-shaft sinuation (Bousquet 1985, 1992) seen in *Pterostichus shulli*, *Pterostichus spathifer*, the following new species(*Pterostichus lolo*), and, to a lesser degree, *Pterostichus smetanai*. The small size and thin body form of *Pterostichus bousqueti* adults are most similar to *Pterostichus campbelli* adults, which occur only west of the Cascade Range in Oregon.

#### Description.

Size medium-small for subgenus, ABL males 8.5–9.5 mm, female 8.9 mm. Body form slightly slender for subgenus (Fig. 1).

*Color.* Dorsal body surface black and shiny, pronotal lateral beads piceous, legs, antennae and palpi piceous.

*Microsculpture*. Head with isodiametric meshes on frons; pronotum with faintly impressed transverse meshes on most of disc, meshes more isodiametric basolaterally; elytra with markedly transverse meshes, faintly iridescent in some areas.

**Figure 3.**
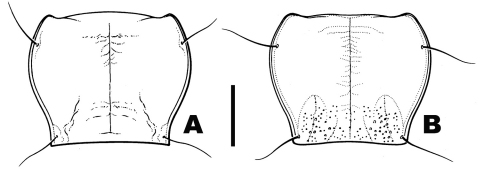
Pronotum, dorsal aspect **A** *Pterostichus (Pseudoferonina) bousqueti* Bergdahl, sp. n. **B** *Pterostichus (Pseudoferonina) lolo* Bergdahl, sp. n. Scale line = 1.0 mm. Line drawings by Go Sato.

*Pronotum*. Fig. 3A. Almost as long as wide, lateral margins subparallel anterior to hind angles, slightly sinuate, hind angles rectangular or slightly acute, lateral margins (in lateral view) bent ventrally near hind angles. Anterior and posterior transverse impressions present but faintly impressed, median line distinctly impressed, almost entire, but not quite extended to anterior and posterior margins in most specimens examined; anterior transverse impression faintly punctulate. Lateral margins finely beaded, posterior margin without margination, anterior margin minutely beaded in medial one-third. Basolateral fovea deep and broadly linear, slightly convergent toward midline anteriorly, not or only faintly punctulate.

*Elytra.*Intervals flat or only slightly convex, but less so than in any other *Pseudoferonina* species.

*Legs.* Male mesotibiae slightly curved apically.

*Abdomen.* Last visible sternite of male with broad, shallow medial indentation, anterior margin of depression not at all carinate, sternite without protuberances; last visible sternite of males with single pair of anal setae, female with two pairs.

**Figure 4.**
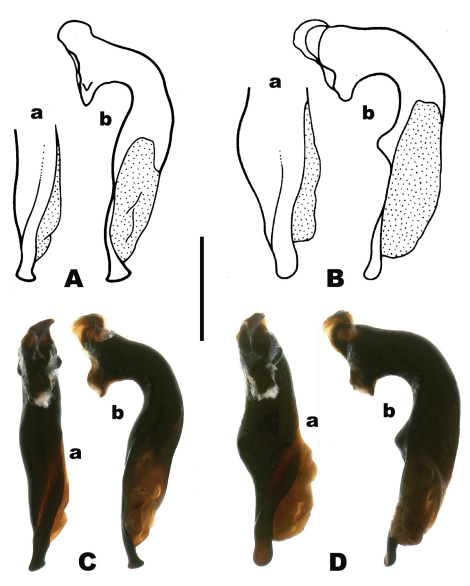
Median lobe of male aedeagus **A–B** line drawings, a = apical region, ventral view; b = left lateral view **C–D** Automontage digital images, a = ventral view, b = left lateral view **A** *Pterostichus (Pseudoferonina) bousqueti* Bergdahl sp. n. **B** *Pterostichus lolo* Bergdahl sp. n. **C** *Pterostichus bousqueti* **D** *Pterostichus lolo*. Scale line = 1.0 mm. Line drawings by G. Sato; digital images by D. H. Kavanaugh.

*Male genitalia*. Median lobe of aedeagus as in Figs 4A and 4C. Apical one-third of median lobe (in ventral view) with lightly sclerotized oblique band extended longitudinally next to slight ridge (Fig. 4C); shaft of median lobe (in ventral view) swollen submedially on left, with shaft gradually tapered from swelling to the apical lamella, ventral margin not sinuate subapically (in lateral view); apical lamella average in length for *Pseudoferonina* species in Idaho, symmetrically hatchet-shaped (in left lateral view). Right paramere short and sharply pointed.

#### Geographic distribution.

**Figure 5.**
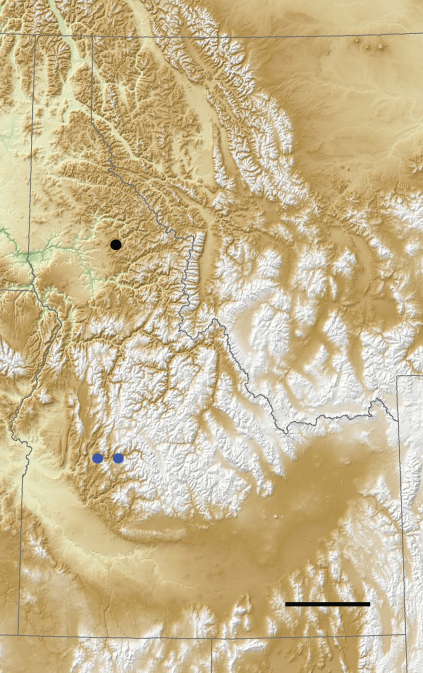
Toporelief map of Idaho illustrating the locations of known localities for *Pterostichus (Pseudoferonina) bousqueti* Bergdahl, sp. n. (blue dots) and *Pterostichus lolo* Bergdahl sp. n. (black dot). Scale line = 100 km.

At this time, *Pterostichus bousqueti* is known only from Pine Flat Creek and Archie Creek near their confluences with the South Fork of the Payette River near Lowman, Boise County, Idaho (Fig. 5). The Payette River is a tributary of the Snake River, which is a major tributary of the Columbia River. To what extent the range of this species is narrowly restricted to this area is not known. Despite sampling many other creeks in this area, JCB has been unable to document any other *Pterostichus bousqueti* populations. Additional areas, if any, in which this species is most likely to occur include the Big Pine, Deadwood, Clear Creek and Middle Fork of the Payette River watersheds to the west of Lowman and east of the North Fork of the Payette River.

#### Habitat.

**Figure 6.**
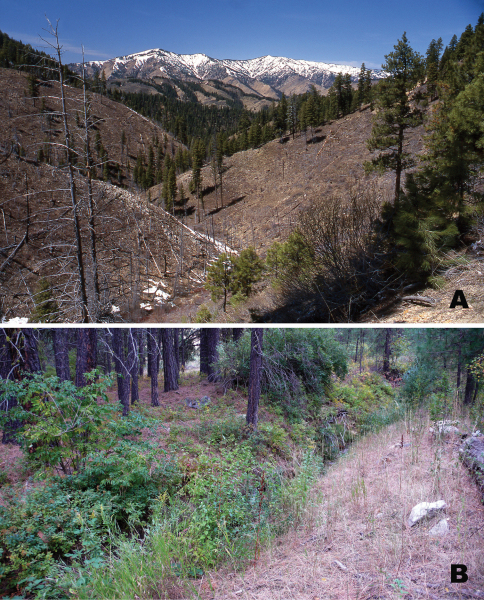
Photographs of habitat for *Pterostichus (Pseudoferonina) bousqueti* Bergdahl, sp. n. **A** View north over South Fork Payette River gorge toward Deadwood Ridge above Lowman, Boise County, Idaho, 5 May 1999 **B** Pine Flat Creek, near Lowman, Boise County, Idaho, 25 September 2008. Photographs by J. C. Bergdahl.

The type locality along the South Fork of the Payette River near Lowman is in a steeply sided, sparsely pine-forested gorge (Fig. 6A) that has experienced intense forest fires. From 1908 to 2000, canopy fires burned more than 50% of the Boise National Forest in central Idaho. Large fires have a huge effect on vegetation, geomorphology, stream hydrographs (Pierce et al. 2004), and undoubtedly *Pterostichus bousqueti* habitat. Fire legacies of this extent on the landscape probably create severe barriers to dispersal of *Pseudoferonina* species. Areas at low to mid-elevations along the South Fork of the Payette River form a small, isolated outlier of the Hot Dry Canyons eco-region of the Idaho Batholith (McGrath et al. 2002). Most of this eco-region is associated with the main stem Salmon River, 145 km (90 miles) to the north. McGrath et al. (2002) describe this eco-region as: unglaciated, deep, precipitous canyons; annual precipitation = 305– 889 mm (lowest in deep canyons); mean temperature: Jan. (min/max) = -8.9/0.6°C, July (min/max) = 8.8/31.7°C (warmer with increasing canyon depth). The vegetation is characterized by open ponderosa pine forest and sagebrush scrub on south facing-slopes, and ponderosa pine/Douglas fir forest on north-facing slopes.

Both Pine Flat Creek (Fig. 6B) and Archie Creek are small, perennial streams descending steep canyon slopes. Their lower sections run thru dry open ponderosa pine forests. At higher elevations on sun and wind-exposed south-facing slopes, the vegetation is primarily open scrub. Pine Flat Creek descends a steep scrubby hillside; pine forest is restricted to lower reaches near the Payette River. Archie Creek descends its watersheds at a gentler grade. Both of these creeks near their confluence with Payette River are only about 1.5 m bank-full-width, and are confined primarily to channels with stabilized vegetated banks. Consequently, there are very few gravel bars on which to search for beetles, even at low water. High water for these creeks most likely occurs March-May, during rapid melt of the snow at higher elevations on adjacent hillsides coincident with wet spring storms. Because the watersheds these creeks service are so small, large thunderstorms and cloudbursts, by themselves, also may cause significant peaks in runoff during summer and fall months after the snow pack has melted at high elevation. In exceptionally dry years, both of these creeks may actually dry up to the extent that even lower sections are without surface-flowing water. However, on 25 September (2008) both creeks had nearly one meter of surface water stream width. Given their small watersheds, it is likely that major sections of both streams, especially high up where slopes are steep and the vegetation scrubby, dry up even in average years.

Other carabid species collected by JCB at or near the type locality include: forest habitat - *Psydrus piceus* LeConte, *Pterostichus protractus* LeConte; streamside habitats - *Platynus brunneomarginatus* (Mannerheim), *Bembidion iridescens* LeConte, *Bembidion kuprianovi* Mannerheim, *Bembidion* sp., *Diplous aterrimus* (Dejean), *Nebria* sp., *Bradycellus californicus* (LeConte), *Bradycellus nigrinus* (Dejean), *Bradycellus nubifer* LeConte. The trout-stream beetle *Amphizoa insolens* LeConte (Coleoptera, Amphizoidae) also occurs along creeks in this area.

#### Phenology and larval ecology.

JCB visited Pine Flat Creek and Archie Creek on two occasions: 05–06 May (1999) and 25 September (2008). In early May, he easily found adults of *Pterostichus bousqueti* at both sites, however in September collecting along these same stream sections on both creeks yielded no specimens. *Pseudoferonina* populations in hot dry regions at low elevations appear to aestivate or go into early hibernation, probably in August. Very few teneral specimens have been collected; however, males with soft, poorly chitinized median lobes are common in late summer and fall. This suggests that adults emerge from pupal chambers in late summer through early fall and overwinter as adults. Presumably they escape winter-spring floods by migrating out of the floodplain to higher positions along stream banks to hibernate in the fall before freeze-up, however they probably do not go very far. Once snow packs melt and daytime temperatures approach 10°C (late March and early April at lower elevations in Idaho), adults again become active on the surface. Given the fact that surface-active *Pseudoferonina* adults are so closely associated with the immediate vicinity of stream margins, the larvae are probably also narrowly restricted to these habitats and may spend a substantial amount of time subsurface at or very near the hyporheic zone.

#### Dispersal power.

All known individuals are brachypterous (flightless) and restricted to the margins of small, isolated, forested streams, so dispersal power of *Pterostichus bousqueti* adults is expected to be extremely low. Like *Pterostichus shulli*, *Pterostichus spathifer* and *Pterostichus lolo* sp. n., the geographic range of *Pterostichus bousqueti* (Fig. 5) appears to be highly restricted.

#### Remarks.

Among Idaho’s *Pseudoferonina* species, *Pterostichus bousqueti* appears to be a member of the *lanei* species-group (with *Pterostichus lanei*), as opposed to the *shulli* species-group (*Pterostichus shulli*, *Pterostichus spathifer* and *Pterostichus lolo* sp. n.). Idaho species in the *shulli* group have a distinct ventral sinuation (bulge and concavity) on the ventral surface of the median lobe of the aedeagus near the middle of the shaft (easily seen in lateral view). Species in the *lanei* group (and *Pterostichus vexatus*) do not have this feature. To what extent this feature, or its absence, is indicative of phylogenetic relationship is presently unknown.

Property ownership in the South Fork Payette River area in vicinity of the type locality is primarily the U.S. Forest Service (Boise National Forest), however according to the Boise National Forest map there are many small in-holdings along the river. Two of these in-holdings include both lower Pine Flat and lower Archie creeks, near where the type specimens were collected. Both of these creeks probably have experienced prospecting or mining activity. There is a small Forest Service campground and hot springs very near Pine Flat Creek on the north bank of the Payette River, but visitors seem to have little impact on the creek.

Because *Pterostichus bousqueti* is known from only two easily identifiable localities, we hope that collectors will exercise restraint when sampling at these sites, and instead focus on discovering new sites for this species in the immediate vicinity, such as in those watersheds listed above.

### Pterostichus (Pseudoferonina) lolo

Bergdahl sp. n.

urn:lsid:zoobank.org:act:50D61144-E2F2-40FB-9A04-FFB9E348C458

http://species-id.net/wiki/Pterostichus_(Pseudoferonina)_lolo

Figs 2 3B 4B, 4D 5 7

#### Type locality.

U.S.A, Idaho, Clearwater County, Cottonwood Creek near the confluence of Orogrande Creek and the North Fork of the Clearwater River, ca. 870 m (2860 ft) elevation, 46.5528°; -115.5522°.

#### Type material.

Holotype*,* a male, deposited in CAS, labeled: “USA: IDAHO, Clearwater Co.; Cottonwood Creek @ USFS Rd 250; Orogrande Ck. Rd.; NF Clearwater R. watershed; elev. ~870 m (2860 ft); 12.5 MI. ESE Headquarters, 3.5 MI. SW of Bungalow; 46°33'10"N, 115°33'08"W; T38N, R7E, S34; 17 July 2009; #73-2009; J. C. Bergdahl, coll.”/ “HOLOTYPE *Pterostichus lolo* Bergdahl designated 2011” [red label]. Paratypes: A total of 22 paratypes from same locale as the holotype (7 males and 5 females from 17 July 2010; 2 males and 9 females from 03 July 2010) deposited in CAS, CMNH, CNC, JCB, OSU, UICM and WSU.

**Figure 7.**
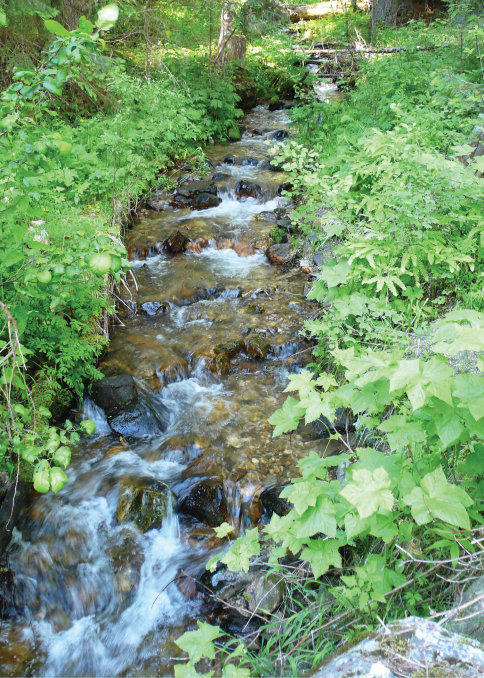
Photographs of habitat for *Pterostichus (Pseudoferonina) lolo* Bergdahl, sp. n. Cottonwood Creek, near Bungalow, Clearwater County, Idaho, 3 July 2010. Photograph by J. C. Bergdahl.

#### Etymology.

The specific epithet, *lolo*, is a noun in apposition, and refers to the Lolo Trail, an ancient hunting and trade route developed by the Nez Perce tribe to cross the Bitterroot Mountains from their homelands on the Clearwater River near Kamiah, Idaho, to the buffalo herds of Montana. The beginning of this trail in its western foothills starts in the upper Lolo River watershed, near the type locality of *Pterostichus lolo*. The Lolo Trail is also the route the Lewis and Clark Expedition took over the Bitterroot Mountains heading west in September 1805 and east in June 1806.

#### Diagnosis.

A *Pterostichus* with the characteristics of members of subgenus *Pseudoferonina* (see Ball 1965; and Bousquet 1999); males easily distinguished from those of all other species of *Pseudoferonina* by the shape of the median lobe of the aedeagus, especially the form of the mid-shaft sinuation and associated convexity (Figs 4B and 4D), which is markedly and abruptly convex, nearly tuberculate (in lateral view), and the shape of the apical lamella (apex), which is broader in ventral view than in *Pterostichus shulli* males (see Bousquet 1992, Fig. 16) and narrower in lateral view than in *Pterostichus spathifer* males (see Bousquet 1992, Fig. 15).

#### Description.

Size medium-large for subgenus, ABL males = 9.5–10.5 mm, females = 9.0–10.3 mm. Body form average for subgenus (Fig. 2).

*Color*. Dorsal surface black and shiny, pronotal lateral beads piceous, antennae and palpi rufous or rufopiceus, femora piceous except distally rufopiceous, tibiae and tarsi rufopiceus or rufous.

*Microsculpture.* Head with faintly impressed isodiametric meshes on frons; pronotum with moderately impressed transverse meshes on most of disc, meshes more isodiametric posteriorly; elytra with moderately impressed transverse meshes and very slight iridescence. *Pronotum.* Fig. 3B. Almost as long as wide, widest point approximately at anterior one-third, lateral margins slightly sinuate in posterior half, almost parallel before hind angles, hind angles slightly obtuse to subrectangular, lateral margins (in lateral view) bent ventrally near hind angles, basal one-quarter of pronotum finely but distinctly punctate. Anterior and posterior transverse impressions faintly or not at all impressed, median longitudinal impression distinctly impressed, nearly extended to anterior and posterior margins in most specimens examined. Lateral margins finely beaded, posterior margin without margination, anterior margin minutely beaded in lateral thirds, without margination in medial one-third. Basolateral fovea deep and broadly linear, slightly convergent toward midline anteriorly, finely but distinctly punctate, areas between basal fovea and lateral margins convex, subcarinate.

*Elytra.* Intervals nearly flat, striae entire, moderately impressed, not or only very faintly punctulate. *Legs.* Male mesotibiae slightly curved apically. *Abdomen.* Last visible sternite of male with broad, shallow medial indentation, anterior margin of depression slightly carinate, sternite without protuberances; last visible sternite of males with single pair of anal setae, female with two pairs.

*Male genitalia*. Median lobe of aedeagus as in Figs 4B and 4D. Apical one-third of median lobe (in ventral view) with lightly sclerotized oblique band extended longitudinally next to slight ridge (Fig. 4Da); shaft of median lobe (in ventral view) markedly swollen submedially on left, with shaft abruptly tapered from swelling to the apical lamella, ventral margin distinctly sinuate subapically (in lateral view) with markedly abrupt, subtuberculate convexity basad of sinuation (Fig. 4Db); apical lamella average in length for *Pseudoferonina* species in Idaho, apex evenly rounded, very slightly swollen in ventral view (Fig. 4Da), narrow and very slightly reflexed ventrally at tip in lateral view (Fig. 4Db). Right paramere as illustrated by Bousquet (1992, Figs 15b and 16b) for *Pterostichus spathifer* and *Bembidion shulli*.

#### Geographic distribution.

Currently, *Pterostichus lolo* is known only from the type locality on Cottonwood Creek (at 870 m), which is a small, low-order tributary of Orogrande Creek, which is itself a tributary of the North Fork of the Clearwater River. In turn, the Clearwater is a tributary of the Snake River, which is a major tributary of the Columbia River. To what extent the range of this species is narrowly restricted to this area is not known. Despite sampling many other creeks in this area over the past decade, JCB has been unable to document any other *Pterostichus lolo* populations.

#### Habitat.

The type locality is in the Clearwater Mountains and Breaks section of the Northern Rockies eco-region (Nesser et al. 1997). This area has mountains of moderate relief with rounded landform, and steep breaklands. Elevations range between ca. 610 and 2070 m (2000 and 6800 ft.). The climate is generally cool, with a significant maritime influence. Summers are warm and dry, winters cool and moist. The soils are mantled by a thick layer of Quaternary volcanic ash and colluvium, underlain by Tertiary granitic rocks of the Idaho Batholith and Precambrian gneiss and schists (McGrath et al. 2002). Annual precipitation is ca. 889– 2032 mm, with 40–50% falling as snow. Snow packs at higher elevations can become very deep and linger late into spring. Rain-on-snow events are common below 1370 m (4500 ft.); January min/max temperatures: -8.3/0.6°C; July min/max temperatures are 7.2/27.2°C.

These mountains have been largely unglaciated and support a highly dissected stream network of ancient narrow valleys and canyons. Drainage density (length of stream/area) is high. The natural vegetation is primarily a very species-rich mosaic of mesic conifer forest, including exceptional conifer species diversity (grand fir, Douglas fir, western red cedar, western hemlock, mountain hemlock, ponderosa pine, lodgepole pine, white pine, western larch, subalpine fir, Englemann spruce and yew). Ponderosa pine/Douglas fir and cedar/hemlock/pine forests occur at lower elevations, and spruce/fir or mountain hemlock forests at higher elevations. Some of the forest types in this area are the best examples of inland temperate rainforest in the Rocky Mountain region (Bergdahl 2008), including western red cedar trees estimated to be more than 3000 years old (Smith and Fischer 1997). The flora of this region has many unique coastal disjunct species and the area is often referred to as the Clearwater Refugium (Brunsfeld et al. 2001, Carstens et al. 2005, Brunsfeld and Sullivan 2006).

Like all other species of *Pseudoferonina*, *Pterostichus lolo* appears to be a habitat specialist, primarily on the wet margins of small, low (0-2) order forest streams descending hillsides in mountainous country. These habitats are often isolated in headwater basins, or on breaks and canyon walls at lower elevations. These beetles are not aquatic, but they are strict hygrophiles. Surface-active adults are usually found within two meters of wet stream channels, primarily within one meter of the strandline and especially right at the water’s edge. When disturbed during warm weather, they will often run into the water and crawl down into the gravel or float away.

Other carabid species collected by JCB at or in the vicinity of the Cottonwood Creek locality include: *Scaphinotus (Pseudonomaretus) regularis* (LeConte), *Scaphinotus (Pseudonomaretus) relictus* (Horn), *Scaphinotus (Pseudonomaretus) merkelli* (Horn), *Zacotus matthewsii* LeConte, *Trechus coloradense* Schaeffer, *Bembidion kuprianovi*, *Bembidion iridescens*, *Bembidion breve* (Motschulsky), *Pterostichus (Hypherpes) ecarinatus* Hatch, *Pterostichus (Leptoferonia) idahoae* Csiki, *Pterostichus (Leptoferonia) beyeri* Van Dyke, *Pterostichus (Pseudoferonina) shulli* (Hatch) and *Pterostichus (Pseudoferonina) vexatus* Bousquet.

#### Phenology and larval ecology.

Adults of *Pterostichus lolo* are probably active from the first warm days of spring after snowmelt to the first days of hard frosts in October. A single pair of adults was observed *in copulo*, on 17 July 2009 [JCB]. *Pterostichus lolo*, and all other *Pseudoferonina* species, are probably “spring breeders” (Bousquet 1986)—that is, they breed early in the active season, larval development takes place in late spring-summer, new adults emerge from pupal chambers in late summer-early fall, and individuals overwinter primarily as immature adults. Males collected in fall are usually immature, suggesting they may die after a single breeding season. These beetles probably vacate streambeds and floodplains in the fall so as to escape winter or spring freshets of high water. Flash floods from thunderstorms during the active season may be a major cause of mortality. The ecology of the larvae is poorly known because they are rarely seen on the surface. The extent to which larvae and adults of *Pseudoferonina* utilize the subterranean hyporheic zone may be substantial. As mentioned above, adults do not hesitate to crawl down into saturated streambed gravels to escape capture. The highest density of adults is often found along small creeks and reaches that have recently experienced small debris slides with rocks embedded in substantial amounts of wet organic mud. Presumably these muddy microhabitats also support the highest larval density and survival.

#### Dispersal power.

All known individuals are brachypterous (flightless) and restricted to the margins of a small, isolated, forested stream, so dispersal power of *Pterostichus lolo* adults is expected to be extremely low. Like *Pterostichus shulli,* *Pterostichus spathifer* and *Pterostichus bousqueti* sp. n., the geographic range of *Pterostichus lolo* (Fig. 5) appears to be highly restricted.

#### Remarks.

Based on the form of the median lobe of the male aedeagus, members of *Pterostichus lolo* appear to be most closely related to those of *Pterostichus shulli* and *Pterostichus spathifer*, (i.e., the *shulli* species-group). It is perhaps significant that both of these species, along with the more distantly related *Pterostichus vexatus*, occur in areas near the single known locality for *Pterostichus lolo*. The North Fork Clearwater River basin has the highest diversity of *Pseudoferonina* species.

Property ownership in the vicinity of type locality is primarily the U.S. Forest Service (Clearwater National Forest). Potlatch Corporation, Bennett Tree Farms, Inc. and the State of Idaho also have significant property ownership in this region. The primary land use impacts in this area are timber harvesting, roads, mining and off-road vehicle use. There is a large, poorly managed campground on U.S. Forest Service property along both sides of Cottonwood Creek at the type locality, with substantial off-road vehicle impacts.

Because *Pterostichus lolo* is known from only one, easily identifiable locality, we hope that collectors will exercise restraint when sampling at this site, and instead focus on discovering new sites for this species in the immediate vicinity.

## Suggested modifications to Bousquet’s key to adults of Pseudoferonina Ball

Bousquet’s key for identification of *Pseudoferonina* adults (Bousquet 1985, modified in 1992) works only for males, and is based mainly on characters of the median lobe of the aedeagus. Although a key that permits the identification of females is highly desirable, we do not yet have sufficient knowledge to construct such a key at this time.

Males of both *Pterostichus bousqueti* and *Pterostichus lolo* key to couplet “3” in Bousquet’s 1985 key. In 1992, he expanded this couplet to accommodate the new species, *Pterostichus spathifer* Bousquet by adding a couplet “3'”. We suggest the following additional modification to accommodate the two new species described here:

**Table:** 

3	Median lobe of aedeagus strongly sinuate on ventral margin behind middle in left lateral view	3a
–	Median lobe of aedeagus not or very slightly sinuate on ventral margin behind middle in left lateral view	3c
3a	Median lobe of aedeagus with apical lamella wide, spoon-like, slightly distorted to right in left lateral view (see Bousquet, 1992, Fig. 15)	*Pterostichus spathifer* Bousquet
–	Median lobe of aedeagus with apical lamella narrower, not spoon-like (see Bousquet, 1992, Fig. 15 and Figs 4B and 4D here)	3b
3b	Median lobe with a broad, evenly arcuate convexity on ventral margin at middle in left lateral view (see Bousquet, 1992, Fig. 16)	*Pterostichus shulli* (Hatch)
–	Median lobe with an abrupt, nearly tuberculate convexity on ventral margin at middle in left lateral view (Figs 4B and 4D)	*Pterostichus lolo* Bergdahl sp. n.
3c	Specimen larger, ABL greater than 10.5 mm; apical lamella of male median lobe nearly parallel-sided, narrow in left lateral view (see Bousquet, 1985, Fig. 9b)	*Pterostichus lanei* Van Dyke
–	Specimen smaller, ABL less than 9.6 mm; apical lamella of male median lobe expanded apically, symmetrically hatchet-shaped in left lateral aspect (Figs 4A and 4C)	*Pterostichus bousqueti* Bergdahl sp. n.

## Discussion

The carabid fauna of the Pacific Northwest (PNW) of North America is highly distinctive, with many endemic and taxonomically isolated taxa, some of which have close affinities (particularly at the generic level) with the southeastern United States or southeast Asia, especially China and Japan (Van Dyke 1940, Kavanaugh 1988). This fauna includes 93 known endemic species and subspecies (Bergdahl 1995). Some of these endemics are restricted to the northern parts of the region, which are assumed to have been fully or largely glaciated in Pleistocene time, as recently as 10,000 years ago (Kavanaugh 1988). There are few, if any, other areas in North America that are known to have been heavily glaciated and yet have such a distinctive fauna with a significant endemic component.

All nine described species in the *Pterostichus* subgenus *Pseudoferonina* are endemic to the PNW. No other group of closely related carabid species includes so many endemics restricted to the region. The large carabid genera *Nebria* Latreille and *Bembidion* Latreille have 13 and 9 species endemic to PNW respectively, but these species represent several subgenera (Bergdahl 1995). Therefore, *Pseudoferonina* may provide opportunities for unique insights into the evolutionary history of the insect fauna of the Pacific Northwest. *Pseudoferonina* includes two species from the Coast Ranges (*Pterostichus humidulus* from northwestern Oregon and southwestern Washington, and *Pterostichus campbelli* from northwestern Oregon), and two from the Cascade Mountains (*Pterostichus smetanai* from southern Washington and *Pterostichus campbelli* from northern Oregon). The other six species (*Pterostichus lanei*, *Pterostichus shulli*, *Pterostichus spathifer*, *Pterostichus lolo*, *Pterostichus bousqueti* and *Pterostichus vexatus*) are found in the interior on the west slope of the Rockies, primarily in the Clearwater, Bitterroot and Salmon River mountains and their foothills in central Idaho. It is clear that the center of diversity of *Pseudoferonina* species is the mountains of central Idaho, especially the Clearwater River basin. This is one of the oldest and most extensive mountain regions in the Pacific Northwest. Given the number of newly described species of *Pseudoferonina* from Idaho and the difficulty of collecting there, it is likely that other species remain to be discovered.

The geographical ranges of all *Pseudoferonina* species remain incompletely known and/or circumscribed. The larvae of all species remain to be recognized and described. Perhaps most importantly, no sister group for *Pseudoferonina* has been identified and phylogenetic relationships among included species have not yet been analyzed. Without a robust hypothesis of relationship among the species, interpretation of the evolution, diversification and history of the group remains impossible. These gaps in our knowledge serve as obstacles to a better understanding of the group and prevent us from answering, at least for the present, interesting questions that should guide future research: What historical and ecological factors are responsible for the diversification of so many localized *Pseudoferonina* species? What is the significance of the disjunct distribution of *Pseudoferonina* in coastal versus inland forest regions? What can an understanding of the phylogenetic and biogeographic history of *Pseudoferonina* tell us about other unique elements of the Pacific Northwest’s insect fauna and their conservation? Hopefully, future discoveries and analyses will help us to answer such questions.

## Supplementary Material

XML Treatment for Pterostichus (Pseudoferonina) bousqueti

XML Treatment for Pterostichus (Pseudoferonina) lolo

## Appendix I

Table of localities. (doi: 10.3897/zookeys.104.1272.app1) File format: Microsoft Office Excel.

Copyright notice: This dataset is made available under the Open Database License (http://opendatacommons.org/licenses/odbl/1.0/). The Open Database License (ODbL) is a license agreement intended to allow users to freely share, modify, and use this Dataset while maintaining this same freedom for others, provided that the original source and author(s) are credited.

## References

[B1] Ball GE (1965) Two new subgenera of *Ptersotichus* Bonelli from western United States, with notes on characteristics and relationships of the subgenera *Paraferonia* Casey and *Feronina* Casey (Coleoptera: Carabidae). Coleopterists Bulletin 36:475-501.

[B2] Bergdahl JC (1995) Ecology and biogeography of the ground beetles (Coleoptera: Carabidae) of the Pacific Northwest of North America. Unpublished report, available through Conservation Biology Center, Spokane, WA, USA.

[B3] Bergdahl JC (2008) Ecology, biogeography and conservation of inland temperate rainforests of the Pacific Northwest of North America: a comprehensive, international perspective. Proceedings of a Conference on British Columbia’s Inland Rainforest – Conservation and Community, 21–23 May 2008 http://web.unbc.ca/~wetbelt/2008-conference-Bergdahl-Ecology-and-Biogeography.html.

[B4] Bonelli FA (1810) Observations entomologiques. Première partie (cicindélètes et portion des carabiques) [with the “Tabula synoptic exhibens genera carabicorum in sections et stirpes deposita”]. Turin, 58 pp + 1 table.

[B5] Bousquet Y (1985) The subgenus *Pseudoferonina* Ball (Coleoptera: Carabidae: *Pterostichus*): description of three new species with a key to all known species. Pan-Pacific Entomologist 61:253-260.

[B6] Bousquet Y (1986) Observations on the life cycle of some species of *Pterostichus* (Coleoptera: Carabidae) occurring in northeastern North America. Le Naturaliste Canadien (revue d’écologie et de systématique)113: 295–307.

[B7] Bousquet Y (1992) Description of new or poorly known species of *Gastrosticta* Casey, 1918, and *Paraferonina* [sic] Ball, 1965 (Coleoptera: Carabidae: *Pterostichus* Bonellli, 1810). Journal of the New York Entomological Society 100:510-521.

[B8] Bousquet Y Larochelle A (1993) Catalogue of the Geadephaga (Coleoptera: Trachypachidae, Rhysodidae, Carabidae including Cicindelini) of America north of Mexico. Memoirs of the Canadian Entomological Society, No. 167, 397 pp.

[B9] Bousquet Y (1999) Supraspecific classification of the Nearctic Pterostichini (Coleoptera: Carabidae). Fabreries, Supplement 9, 292 pp.

[B10] Brunsfled SJ Sullivan J (2006) A multi-compartmented glacial refugium in the northern Rocky Mountains: evidence from the phylogeography of *Cardamine constancei* (Brassicacceae). Conservation Genetics 6:895-904. doi: 10.1007/s10592-005-9076-7

[B11] Brunsfled SJ Sullivan J Soltis DE Soltis PS (2001) Comparative phylogeography of northwestern North America: a synthesis. In: Silvertown J, Antonovis J (Eds) Integrating ecology and evolution in a spatial context. Blackwell Publishing, Williston, VT, 319–339.

[B12] Carstens BC Brunsfeld SJ Demboski JR Good JM Sullivan J (2005) Investigating the evolutionary history of the Pacific Northwest mesic forest ecosystem: hypothesis testing within a comparative phylogeographic framework. Evolution 59:1639-1652. doi: 10.1554/04-661.1 16331838

[B13] Darlington PJ (1971) The carabid beetles of New Guinea. Part IV. General considerations; analysis and history of fauna; taxonomic supplement. Bulletin of the Museum of Comparative Zoology 142:129-337.

[B14] Dupuis L Friele P (2006) The distribution of the Rocky Mountain tailed frog (*Ascaphus montanus*) in relation to the fluvial system: implications for management and conservation. Ecological Research 21:489-502. doi: 10.1007/s11284-006-0147-0

[B15] Hatch MH (1949) Studies on the Coleoptera of the Pacific Northwest III: Carabidae: Harpalinae. Bulletin of the Brooklyn Entomological Society 44:80-88.

[B16] Hatch MH (1953) The beetles of the Pacific Northwest. Part I: Introduction and Adephaga. University of Washington Publications in Biology 16, 340 pp.

[B17] Kavanaugh DH (1988) The insect fauna of the Pacific Northwest coast of North America: present patterns and affinities and their origins. In: Downes JA, Kavanaugh DH (Eds) Origins of the North American insect fauna. Memoirs of the Entomological Society of Canada, No. 144, 125–150.

[B18] Kavanaugh DH LaBonte JR. (2006) *Pterostichus brachylobus* Kavanaugh and LaBonte, a new species of the carabid subgenus *Hypherpes* Chaudoir, 8138, from the central coast of Oregon (Insecta: Coleoptera: Carabidae: Pterostichini). Proceedings of the California Academy of Sciences (Series 4) 57:215-223.

[B19] LaBonte JR (2006) *Pterostichus lattini* LaBonte, a new species of carabid beetle (Coleoptera: Carabidae: Pterostichini) from Oregon. Proceedings of the California Academy of Sciences (Series 4) 57:203-213.

[B20] Lindroth CH (1966) The ground-beetles of Canada and Alaska, Part 4. Opuscula Entomologica, Supplement 29, 409–648.

[B21] McGrath CL Woods AJ Omernik JM Bryce SA Edmondson M Nesser JA Shelden J Crawford RC Comstock JA Plocher MD (2002) Ecoregions of Idaho (color poster with maps, descriptive text, summary table, and photographs). U.S. Geological Survey, Reston, VA, map scale=1:1,350,000.

[B22] Nesser JA Ford GL Maynard CL Page-Dumroese DS (1997) Ecological units of the Northern Region – subsections (map and text). U.S .Department of Agriculture, Forest Service, Intermountain Research Station, Ogden, UT, General Technical Report INT-GTR-369, 88 pp, map scale= 1:3,500,000.

[B23] Pierce JL Meyers GA Jull AJT (2004) Fire-induced erosion and millennial scale climate change in northern ponderosa pine forests. Nature 432:87-90. doi: 10.1038/nature03058 15525985

[B24] Smith JK Fischer WC (1997) Fire ecology of the forest habitat types of northern Idaho. U.S. Department of Agriculture, Forest Service, Intermountain Research Station, Ogden, UT, General Technical Report INT-GTR-363, 142 pp.

[B25] Strahler AN (1957) A Quantitative analysis of watershed morphology. American Geophysical Union Transactions 38:913-920.

[B26] Van Dyke EC (1925-26) New species of Carabidae in the subfamily Harpalinae, chiefly from western North America. Pan-Pacific Entomologist 2: 65–76 [1925], 113–126 [1926].

[B27] Van Dyke EC (1940) The origin and distribution of the coleopterous insect fauna of North America. In: Proceedings of the 6th Pacific Science Congress, Pacific Science Association. University California Press, Berkeley, CA, 255–268.

[B28] Van Dyke EC (1943) New species and subspecies of North American Carabidae. Pan-Pacific Entomologist 19:17-30.

